# A Tale of Overlapping Autoimmune Serologies: Diagnosing Hydralazine-Induced Antineutrophilic Cytoplasmic Antibody (ANCA)-Associated Vasculitis (AAV) Related With Isolated Pulmonary Involvement

**DOI:** 10.7759/cureus.89547

**Published:** 2025-08-07

**Authors:** Pratikshya Thapa, Indresh Yadav, Julia Vinagolu-Baur

**Affiliations:** 1 Department of Internal Medicine, Nuvance Health, Poughkeepsie, USA; 2 Department of Infectious Diseases, University of Louisville School of Medicine, Louisville, USA; 3 Norton College of Medicine, SUNY Upstate Medical University, Syracuse, USA

**Keywords:** anca-associated vasculitis, hydralazine toxicity, overlapping autoimmune serologies, pleural effusion, pulmonary vasculitis, systemic lupus erythematosus

## Abstract

Hydralazine is an antihypertensive that can induce immune-related adverse effects, such as hydralazine-induced lupus and hydralazine-induced antineutrophilic cytoplasmic antibody (ANCA)-associated vasculitis (AAV). AAV involves necrotizing inflammation of small blood vessels, manifesting as fever, malaise, arthralgia, and myalgia, potentially leading to organ failure. Diagnosis includes clinical evaluation, serological testing for ANCA, and histopathological examination, confirmed by necrotizing granulomatous inflammation in affected tissues. This case report presents a rare presentation of hydralazine-induced AAV limited to the lungs, with isolated pleural involvement. A 57-year-old Caucasian male with hypertension, severe diastolic dysfunction, and end-stage renal disease undergoing dialysis presented with dyspnea, cough, and fatigue. Examination revealed bilateral lung crackles and peripheral edema. Chest computed tomography (CT) revealed a pleural effusion. Despite dialysis and thoracentesis, the patient showed no improvement, leading to pleural fluid analysis, which revealed an exudative pattern with reactive mesothelial cells. An autoimmune workup revealed elevated levels of antinuclear antibodies (ANA), anti-dsDNA, anti-histones, and ANCA. This presents a diagnostic dilemma: evolving systemic lupus erythematosus (SLE) or drug-induced vasculitis. Studies have highlighted that ANCA-positive patients may display serological features that overlap with SLE. Using the 2019 American College of Rheumatology (ACR) SLE criteria and biopsy results, we ruled out certain conditions and ultimately diagnosed the patient with chronic hydralazine-induced AAV. The patient's condition improved after hydralazine discontinuation and initiation of corticosteroid therapy. Pleural effusion can be an initial sign of AAV, and clinicians should be vigilant, especially in patients with end-stage renal disease who are unresponsive to dialysis.

## Introduction

Antineutrophil cytoplasmic antibody (ANCA)-associated vasculitis (AAV) encompasses a group of severe small-vessel vasculitis characterized by necrotizing inflammation of the blood vessels [1]. ANCAs are autoantibodies that are directed against components of neutrophil granules, most commonly myeloperoxidase (MPO) and proteinase 3 (PR3) - their presence helps distinguish AAV from other autoimmune conditions by guiding serological diagnosis. AAV can involve various organs, including the upper and lower respiratory tracts, kidneys, and, in rare cases, other organs such as the liver [1]. While the etiology of idiopathic ANCA vasculitis is unclear, certain medications, including hydralazine, have been associated with drug-induced AAV [2]. Hydralazine, an antihypertensive drug, is also used to reduce the afterload during chronic heart failure treatment [3]. However, hydralazine-induced lupus can trigger immune-related adverse effects (affecting 7-13% of users) and, less frequently, hydralazine-induced AAV [1,3]. Although both are immune-mediated conditions triggered by hydralazine, hydralazine-induced lupus typically presents with positive ANA and anti-histone antibodies without ANCA positivity, whereas hydralazine-induced AAV involves ANCAs (especially MPO or PR3) and often leads to small-vessel vasculitis, making the distinction between the two a key diagnostic dilemma to be solved in both clinical diagnosis and management. Early diagnosis and treatment are essential to prevent irreversible organ damage and effectively manage the disease [4]. This case report highlights an unusual presentation of hydralazine-induced ANCA vasculitis limited to the lungs, with isolated pleural involvement, challenging traditional diagnostic paradigms.

## Case presentation

A 57-year-old Caucasian male presented to the emergency department with significant respiratory distress, including shortness of breath, cough, fatigue, and loss of appetite. He was hypoxic to 82% SpO_2_ and required 10 L of oxygen via nasal cannula. Physical examination revealed an obese, tachypneic male with bilateral lung crackles and peripheral edema. His medical history included hypertension, severe diastolic dysfunction, end-stage renal disease on maintenance dialysis, and recurrent acute hypoxic respiratory failure due to bilateral pleural effusions.

Initial labs showed elevated inflammatory markers: erythrocyte sedimentation rate (ESR) of >140 mm/h, C-reactive protein (CRP) of 126 mg/dL, normal WBC count of 6.3 x 10^9^/L, and serum creatinine of 4.24 mg/dL (baseline: 4.0 mg/dL). His estimated glomerular filtration rate (GFR) was 16 mL/min/1.73 m^2^, and blood urea nitrogen (BUN) was 53 mg/dL. Urinalysis indicated significant protein, specific gravity of 1.011, and 51-100 RBCs per high-powered field (HPF) (Tables 1-2).

**Table 1:** Pertinent laboratory findings with reference ranges CBC-WBC: Complete Blood Count-White Blood Cells; CRP: C-Reactive Protein; ANA: Antinuclear Antibody; DNA(ds) Ab: Double-Stranded DNA Antibody; IFA: Immunofluorescence Assay; ANCA: Anti-Neutrophil Cytoplasmic Antibody; p-ANCA: Perinuclear Anti-Neutrophil Cytoplasmic Antibody; MPO Ab: Myeloperoxidase Antibody; PR3 Ab: Proteinase 3 Antibody; RBC/HPF: Red Blood Cells per High Power Field; ESR: Erythrocyte Sedimentation Rate; C3/C4: Complement Component 3/Complement Component 4; IU/mL: International Units per Milliliter; AU/mL: Arbitrary Units per Milliliter; Cyc Cit Peptide: Cyclic Citrullinated Peptide Antibody; SSA Ab: Anti-Sjögren’s Syndrome Antibody A; SSB Ab: Anti-Sjögren’s Syndrome Antibody B; Scl-70 Ab: Scleroderma (Topoisomerase I) Antibody; SM/RNP: Smith/Ribonucleoprotein Antibody; MDA-5: Melanoma Differentiation-Associated Protein 5 Antibody; Mi-2 Ab: Mi-2 (Myositis-Specific) Antibody; NXP Ab: Nuclear Matrix Protein Antibody; EBV – Epstein-Barr Virus; TB QuantiFERON: Tuberculosis QuantiFERON Test

Investigation	Result	Reference Value
CBC-WBC	6.3 x 10^9^/L	3.5-10.0 x 10^9^/L
Chemistry Panel-Creatinine	4.24 mg/dL - within baseline	0.67-1.23 mg/dL
Urinalysis: Proteinuria	200 mg/dL	Negative
Urinalysis: Hematuria	51-100 RBC/HPF	0-2 RBC/HPF
ESR	>140 mm/hr	0-20 mm/hr
CRP	126 mg/dL	<1 mg/dL
ANA	1:1280	<1:40 = negative, 1:40-1:80 = Low antibody level, >1: 80 = Elevated Antibody Level
DNA(ds)Ab Crithidia	80 IU/mL	0-24 (HI)
DNA(ds) Ab Crith IFA	1:40	<1:10
Anti-Histone antibodies	>7 units	0.0-0.9 units
ANCA	p-ANCA	Negative
ANCA titer	>1:1280 (HI)	<1:1280
MPO Ab	73 AU/mL	0-19 AU/mL
PR3 Ab	108 AU/mL	0-19 AU/mL
Complement C3	86 mg/dL	90-180 mg/dL
Complement C4	10 mg/dL	10-40 mg/dL
Rheumatoid factor	11 IU/mL	<=13 IU/mL
Cyc Cit peptide	<0.5 unit/mL	0.0-2.9 unit/mL
SSA Ab	<0.2 AI	0.0-0.9
SSB Ab	<0.2 AI	0.0-0.9
Scl 70 Ab	5 AU/mL	0-40 AU/mL
SM/RNP antibody	4 units	0-19 units
Anticentromere	79 AU/mL	0-40 AU/mL
Hepatitis panel	Negative	Negative
EBV	Negative	Negative
Parvo virus	Negative	Negative
Tb QuantiFERON test	Negative	Negative
MDA-5	Negative	Negative
Mi-2 Ab	Negative	Negative
NXP Ab	Negative	Negative
Aldolase	3.6 unit/L	1.2-7.6 unit/L

**Table 2:** Light criteria of pleural fluid analysis showing an exudative pattern LDH: Lactate Dehydrogenase; g/dL: Grams per Deciliter; unit/L: Units per Liter

Criteria	Finding
Color	Straw appearing
Pleural Protein	3.1 g/dL
Pleural LDH	137 unit/L
Serum LDH	188 unit/L
Total Protein	5.8 g/dL
Pleural:Serum LDH	137/156 (0.87)
Pleural:Serum Protein	3.1/5.8 (0.53)

**Figure 1:**
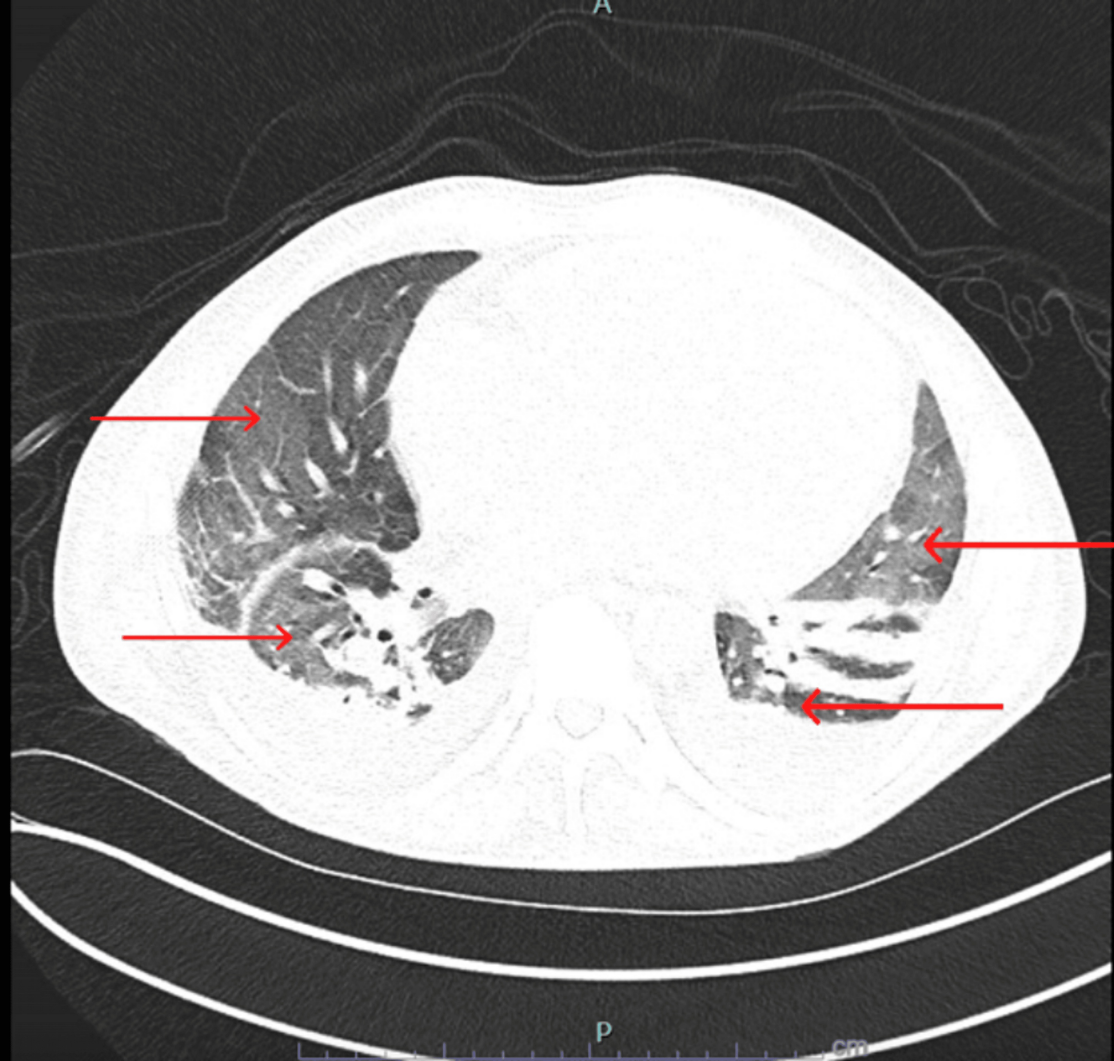
CT chest showing bilateral pleural effusions and patchy ground-glass infiltrates

**Figure 2:**
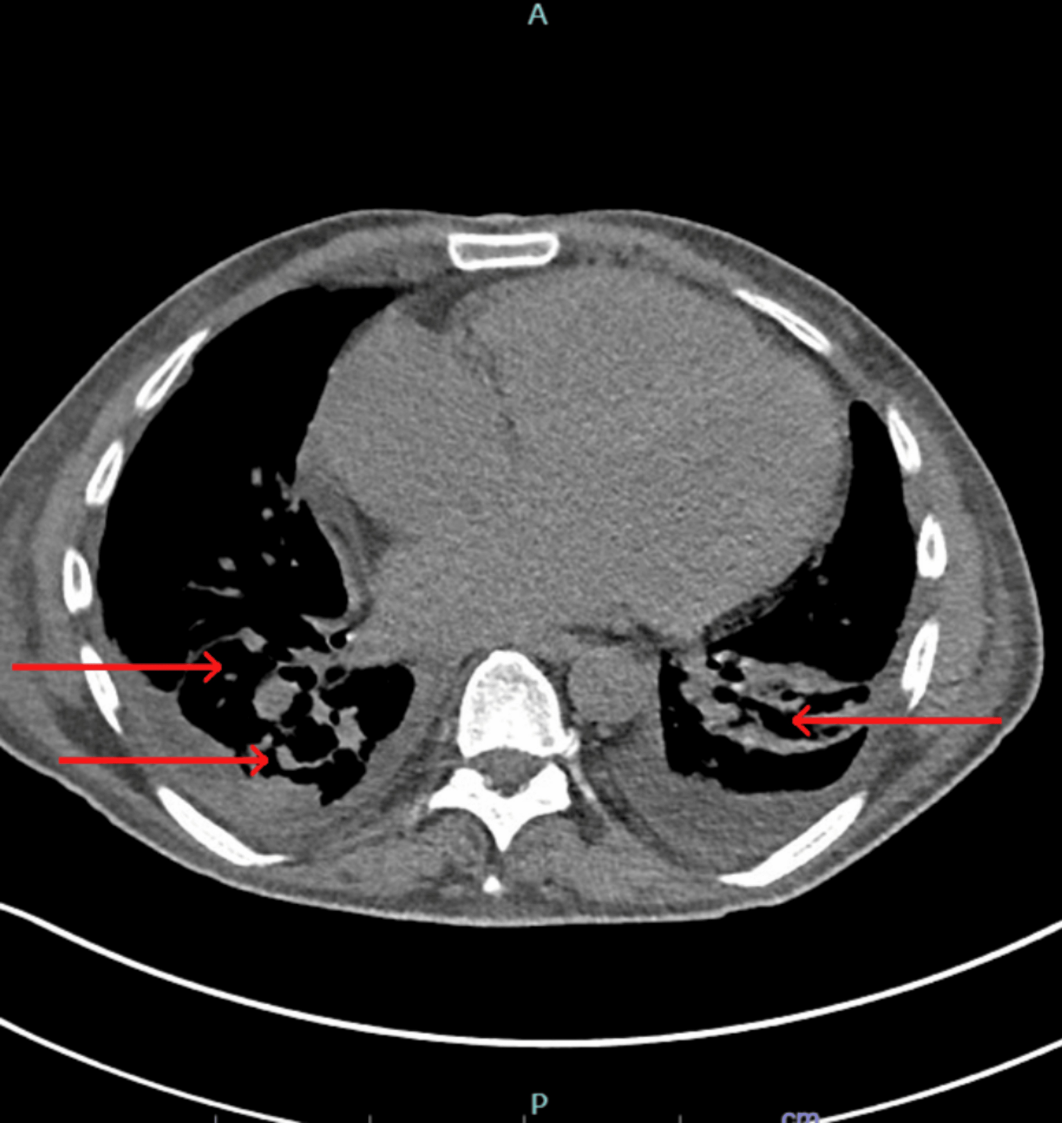
Non-contrast CT chest showing bilateral pleural effusions and patchy ground-glass infiltrates

Chest radiography (CXR) revealed bilateral pleural effusion, increased pulmonary venous congestion, and a diffuse interstitial prominence (Figure 1). Non-contrast chest CT showed moderate bilateral pleural effusion and patchy ground-glass infiltrates (Figure 2).

Due to worsening status, he underwent multiple dialyses without improvement. Repeat CXR showed no changes. He underwent bilateral thoracentesis, chest tube placement, and scheduled dialysis, which resulted in mild improvement. Pleural fluid analysis revealed an exudative pattern that was negative for malignant cells with scattered reactive mesothelial cells, macrophages, and mixed inflammatory cells. The autoimmune workup showed high ANA (1:1280 homogenous), positive ANCA markers (p-ANCA of 1:1280, MPO of 90 AU/mL, PR-3 of 99 AU/mL), anti-centromere antibodies (79 AU/mL), anti-deoxyribonuclease antibodies (<86), and histone IgG g>7 units. The complement levels were low (C3: 76 mg/dL; C4: 8 mg/dL) (Tables 1-2). The workup was negative for scleroderma, Sjogren's syndrome, multiple myeloma, myasthenia gravis, anti-glomerular basement membrane antibodies, and tuberculosis (TB). Abdominal and pelvic CT showed atrophic kidneys without hydronephrosis, and renal biopsy indicated diffuse global glomerulosclerosis consistent with end-stage kidney disease due to diabetes, tubular atrophy, and interstitial fibrosis.

Suspecting ANCA vasculitis potentially associated with drug-induced positivity, hydralazine, which was used since 2015, was discontinued. High-dose steroid therapy was initiated, leading to reduced oxygen requirements and improved clinical outcomes. Scheduled dialysis continued for end-stage renal disease. The patient was discharged on a tapering steroid regimen with a referral to rheumatology for further management. In subsequent outpatient care involving rheumatology and pulmonology specialists, he received rituximab and continued oral prednisone. Discussions regarding obtaining a lung tissue biopsy specimen are ongoing.

## Discussion

Systemic AAV are categorized based on vascular inflammation distribution and presence of granulomatosis and asthma as GPA (Wegener's granulomatosis), EGPA (Churg-Strauss syndrome), and MPA. AAV are rare diseases, with a worldwide incidence of 13-20 cases per million individuals per year and prevalence of 46-184 cases per million [5]. The occurrence and prevalence of AAV are increasing, likely due to enhanced understanding. Initially, AAV can impact one or multiple organs [5]. A fourth subtype, renal-limited AAV, is recognized, but no lungs/pleura-limited limited has been classified [5].

The pathogenesis of AAV is complex and not fully understood, but a combination of genetic susceptibility and environmental triggers (e.g., infections), drugs (e.g., hydralazine), and silica, is believed to play a role. Notable risk factors for developing hydralazine-induced ANCA-associated vasculitis include a cumulative dose exceeding 100 g, prolonged therapy duration, female sex, slow hepatic acetylation, the presence of the null C4 gene, and a history of thyroid disease [1,3]. The exact mechanism of hydralazine-induced AAV is not completely understood, but it is believed that hydralazine causes neutrophils to express MPO and PR3 [6]. Activated neutrophils release reactive oxygen species (ROS) and form extracellular traps (NETs), leading to apoptosis [7]. This releases MPO and PR3 into the extracellular space, processed by dendritic cells, releasing TGF-β and IL-6, differentiating naive T cells into Th17 cells, which produce IL-17, prompting macrophages to release TNF-α and IL-1β, significant priming agents [6]. Th2 cells assist B cells in producing ANCAs, further activating neutrophils [8]. The activation of the alternative complement pathway produces C5a, which also primes neutrophils and contributes to vascular injury [3,6].

The diagnostic approach for AAV typically involves clinical evaluation, serological testing for ANCA (especially anti-PR3, MPO antibodies), and histopathological examination of biopsy specimens [7]. A definitive diagnosis is confirmed by evidence of necrotizing granulomatous inflammation and vasculitis in affected tissues, often with positive ANCA findings [6]. In addition to ANCA antibodies, AAV can also lead to positive serologies for markers such as ANA, anti-double-stranded DNA, and anti-histone antibodies [1,3]. A study conducted by Kumar et al. involving 323 patients with AAV identified 12 patients who exhibited serological features resembling those of systemic lupus erythematosus (SLE), including positive ANA, dsDNA, and anti-histone antibodies, along with low complement levels [8]. Similarly, a literature review by Doughem et al. of 35 patients with lung-kidney syndrome resulting from hydralazine-induced AAV found positive ANA, anti-MPO, and anti-histone antibodies as well [1].

In our case, serological tests overlapped with lupus, and to differentiate the diagnosis, our evaluation focused on both the clinical features and serological findings. The presence of a positive ANA titer, ds-DNA along with positive ANCA, and other lupus anticoagulants, suggested the possibility of SLE mimickers rather than true SLE, using the 2019 American College of Rheumatology (ACR) SLE criteria. A renal biopsy is essential for diagnosing hydralazine-induced AAV and differentiating it from hydralazine-induced lupus, which usually shows pauci-immune glomerulonephritis. However, our patient's renal biopsy results were unremarkable, indicating diabetic nephropathy.

Treatment involves the induction and maintenance phases [6]. Induction typically involves high-dose glucocorticoids (GCs) with rituximab or cyclophosphamide [6]. Our patient was treated with high-dose steroid therapy, which decreased oxygen requirements, and was discharged on a tapering steroid regimen with a referral to rheumatology for further management. In subsequent outpatient care, involving rheumatology and pulmonology specialists, he received rituximab and continued oral prednisone. Major causes of mortality include infections and kidney failure, particularly in the first year post diagnosis, as untreated AAV has a 90% mortality rate after one year [6].

The prognosis is variable, ranging from complete recovery to lifelong immunosuppression. The cause of these differences is likely multifactorial [7]. Therefore, further investigation is needed to understand the underlying mechanisms, associated risk factors, and optimal management of rare but significant complications related to hydralazine. Further research is needed to determine whether it is advisable to postpone hydralazine treatment in patients with or without heart failure, given its potential to lead to severe autoimmune diseases [3].

## Conclusions

This case highlights the challenges in diagnosing hydralazine-induced AAV affecting the lungs and pleura, especially with comorbidities such as renal and cardiac failure and overlapping autoimmune serologies. This underscores the need for a detailed medication history, thorough autoimmune workup, and biopsy for an accurate diagnosis and management. To improve early recognition of hydralazine-induced AAV in clinical practice, it is imperative to consider key diagnostic red flags, including a history of hydralazine use, and dual positivity for anti-MPO and anti-PR3 antibodies to aid pattern recognition in clinical practice. The key steps include discontinuing hydralazine and initiating immunosuppressive therapy. This case emphasizes the need for research on alternative heart failure and antihypertensive medications. Additionally, it contributes to the existing literature by providing insight into serological overlap with SLE and emphasizing the importance of biopsy findings in establishing a correct diagnosis, deepening our understanding of hydralazine effects and AAV management.
